# Inhibitors of Histone Deacetylases Are Weak Activators of the* FMR1* Gene in Fragile X Syndrome Cell Lines

**DOI:** 10.1155/2017/3582601

**Published:** 2017-10-25

**Authors:** Alexander A. Dolskiy, Vladimir O. Pustylnyak, Andrey A. Yarushkin, Natalya A. Lemskaya, Dmitry V. Yudkin

**Affiliations:** ^1^Chromosome Pathology Group, Institute of Molecular and Cellular Biology, Siberian Branch of the Russian Academy of Sciences, Novosibirsk 630090, Russia; ^2^Novosibirsk State University, Novosibirsk 630090, Russia; ^3^The Institute of Molecular Biology and Biophysics, Novosibirsk 630117, Russia

## Abstract

Fragile X syndrome is the most common cause of inherited intellectual disability in humans. It is a result of CGG repeat expansion in the 5′ untranslated region (5′ UTR) of the* FMR1* gene. This gene encodes the FMRP protein that is involved in neuronal development. Repeat expansion leads to heterochromatinization of the promoter, gene silencing, and the subsequent absence of FMRP. To date, there is no specific therapy for the syndrome. All treatments in clinic practice provide symptomatic therapy. The development of drug therapy for Fragile X syndrome treatment is connected with the search for inhibitors of enzymes that are responsible for heterochromatinization. Here, we report a weak transcriptional activity of the* FMR1* gene and the absence of FMRP protein after Fragile X syndrome cell lines treatment with two FDA approved inhibitors of histone deacetylases, romidepsin and vorinostat. We demonstrate that romidepsin, an inhibitor of class I histone deacetylases, does not activate* FMR1* expression in patient cell cultures, whereas vorinostat, an inhibitor of classes I and II histone deacetylases, activates a low level of* FMR1* expression in some patient cell lines.

## 1. Introduction

Fragile X syndrome (FXS) is the main cause of inherited intellectual disability in humans caused by CGG repeat expansion in the 5′ UTR of the* FMR1* gene. The normal allele contains less than 55 triplets. FXS corresponds to a fully mutated allele that contains greater than 200 CGG triplets. Expansion leads to methylation of the* FMR1* promoter and of the expanded CGG triplet, resulting in silencing of gene expression. FMR1 encodes the FMRP protein that is involved in neuronal development [1]. One of the directions of syndrome treatment developing is symptomatic therapy. Some symptoms can be suppressed by Gp1mGlu receptor antagonists or by agonists of *γ*-aminobutyric acid receptors [2]. Traditional clinical practice involves patient treatment with folic acid [3]. Additional reports describe the effect of minocycline in FXS patients [1]. All described methods have a similar restriction: the therapy does not restore* FMR1* gene expression. The search for drugs that activate the* FMR1* gene is thought to be an important scientific direction.

Heterochromatinization includes DNA methylation and histone modifications. Some authors reported DNA methylation followed by histone modifications, such as changes in lysine in the N-terminus of histones by histone acetyltransferases [4, 5]. The most important histone modifications are changes of the N-terminus. High transcription levels coincide with high acetylation of histones H3 and H4 at the N-terminus, whereas silenced transcription is noted with low acetylation [5]. Repeat expansion in the* FMR1* leads to deacetylation of histones H3 and H4 in the locus. Moreover, additional markers of silenced chromatin can be observed in the region [6]. However, it has been shown that a decreased transcriptional activity of the* FMR1* gene in embryonic cells HESC depends on the modification of histones without DNA methylation [7].

FXS therapy development involves the search for chemicals that inhibit enzymes responsible for heterochromatinization. One method involves DNA methyltransferase (DNMT) inhibition in FXS cell lines with 5-aza-2-deoxycytidine (5-azadC). This drug reactivates* FMR1* expression in FXS cell lines [8, 9]. Additional studies used inhibitors of other chromatin modification enzymes, namely, histone deacetylases (HDACs). Three HDACs inhibitors, 4-phenylbutyrate, sodium butyrate, and trichostatin A (TSA), have apparent but modest reactivating effects on the* FMR1* gene in FXS cells. All studied inhibitors are not applicable for drug development given their low effect [10].

To date, three of HDAC inhibitors (vorinostat, belinostat, and romidepsin) are approved by the FDA for human treatment as anticancer drugs. Romidepsin is dipeptide that inhibits class I HDACs. Vorinostat and belinostat are hydroxamic acid derivatives that inhibit class I and II HDACs [11, 12].

Here, we present study of the ability of romidepsin and vorinostat to activate* FMR1* gene expression in FXS patient cell lines.

## 2. Materials and Methods

### 2.1. Cell Cultures

All cell lines in the study are immortalized B-lymphocytes. The full mutation cell line GM04025 from the Coriell Cell Repository (Coriell Institute, USA) has a repeat size of 645 triplets and a methylated promoter [13, 14]. Another full mutation cell line, CPG7, is from the IMCB SB RAS cell repository. This cell line has a methylated promoter and 11.2% of FRAXA fragility, which corresponds to FXS. Two control cell lines GM06865 and GM06895 are from the Coriell Cell Repository and carrying less than 30 repeats and an unmethylated* FMR1* promoter [15]. Cells were cultivated in RPMI 1640 GlutaMAX medium (Gibco, USA) with 15% fetal bovine serum (Gibco, USA) and antibiotics.

### 2.2. Drug Treatment

The 10 mM 5-azadC (PubChem CID 451668) (Sigma-Aldrich, USA) stock solutions were prepared in sterile water and stored at −20°C in aliquots. The following stock solutions were prepared in sterile 100% DMSO and stored at −20°C: 0.5 mM trichostatin A (PubChem CID 444732) (Sigma-Aldrich, USA); 15 *μ*M, 50 *μ*M, and 250 *μ*M romidepsin (PubChem CID 5352062) (Toronto Research Chemicals, Canada); 250 *μ*M, 0.5 mM, and 5 mM vorinostat (SAHA) (PubChem CID 5311) (Sigma- Aldrich, USA).

In the case of 5-azadC treatment, cells were counted, split, and seeded at the initial concentration of 5*∗*10^5^ cells/ml in a total volume of 10 ml per flask. Immediately before use, 10 *μ*l of the 10 mM 5-azadC stock solution was thawed and added daily to the flasks and thoroughly resuspended (final concentration 10 *μ*M, as described previously [10]), whereas a control flask was left untreated. Then, 2*∗*10^6^ cells were harvested after 8 days from the start of treatment for RNA extraction, washed twice with 1х PBS solution, and stored at −70°C [10].

In the case of romidepsin and vorinostat treatment, cells were counted, split, and seeded at the initial concentration of 7*∗*10^5^ cells/ml in a total volume of 15 ml per flask. Immediately before use, 15 *μ*l of the 0.5 mM trichostatin A stock solution was thawed, added once to the flasks, and thoroughly resuspended (final concentration 0.5 *μ*M). The concentration of romidepsin and vorinostat was determined experimentally. The controls were B lymphocyte cell lines without treatment or treated with 15 *μ*l 100% DMSO (solvent for these agents). Briefly, 5*∗*10^5^ сells were harvested after 72 hours from the start of treatment for RNA and protein extraction, washed twice with 1х PBS solution, and stored at −70°C.

### 2.3. Viability of Cell Cultures

Viability of cell cultures was determined by Trypan blue staining followed by counting with a hemocytometer. Viability was determined as *X* = (1 − *a*/*b*)*∗*100, where *X* is the viability of cell culture, *a* is the number of stained cells, and *b* is the total number of cells.

### 2.4. RNA Purification and RT-PCR

RNA was purified from cell cultures using CCR-100 RNA purification kit (BioSilica, Russia) followed by reverse transcription with iScript™ Select cDNA Synthesis Kit (BioRad, USA). Real-time PCR was performed with iQ™ SYBR® Green Supermix (BioRad, USA) on a CFX96 Touch™ Real-Time PCR Detection System (BioRad, USA). Primers and normalization were used as described before [16]. Normalization of the results was performed using the level of expression of the gene* FMR1* and GAPDH in cell line GM06895 as described earlier and all X-fold changes presented as values relative to GM06895 [16, 17]. Statistical analysis was performed as described earlier [18]. Statistical significance of differences was calculated by two-sample *t*-test using GraphPad Prism software (CA, USA). Each sample consisted of three biological replicates in a single experiment. Differences were significant if *P* < 0.05, where *P* is type I error.

### 2.5. Preparation of Proteins Extract

Each sample of cells was suspended in 100 *μ*l lysis buffer (50 mM Tris-HCl, pH 8; 150 mM NaCl; 1% NP-40; 0.1% SDS) supplemented with protease inhibitor (Pierce™ Protease Inhibitor Mini Tablets, Thermo Fisher). The resulting homogenates were incubated on ice for 30 min and centrifuged at 5000*g* for 10 min to remove insoluble precipitates. Protein concentrations in samples were determined with the Pierce™ BCA Protein Assay Kit (Thermo Fisher) according to the manufacturer's protocol. All samples were diluted with water to the same protein concentration. Protein extracts were collected and stored at −80°C.

### 2.6. SDS-PAGE Electrophoresis and Western Blot

Protein samples were loaded in each lane, separated on a 10% SDS-PAGE, and transferred to a PVDF Transfer Membrane (Thermo Fisher). Membranes were stained with Ponceau S to verify loading and transfer efficiency. FMRP and GAPDH were detected by anti-FMRP (ab130165, Abcam) and anti-GAPDH (ab9485, Abcam, USA) primary antibodies. Secondary antibodies were goat anti-mouse IgG Fc (A16084, Life Technologies) and goat anti-rabbit HRP (AP187P, EMD Millipore), respectively.

## 3. Results

### 3.1. Cell Lines Characterization

Cell lines used for drug testing were carriers of a normal human karyotype. Karyotypic analysis of cell lines tested in our research revealed 2*n* = 46 for each cell line. Analysis of* FMR1* expression in all cell lines was carried out as described previously [17]. All expression levels with or without 5-azadC treatment [8] correspond to expected levels (Table 1(a)).

### 3.2. Inhibitors Concentration Selection

Optimal concentrations of romidepsin and vorinostat were selected based on previously published works. Romidepsin was used for function assays, and the optimal concentration ranged from 10 to 500 nM [19]. Vorinostat was used for functional assays, and the optimal concentration ranged from 100 nM to 5 *μ*M [20]. Thus, we tested reactivating activity of romidepsin using concentrations ranging from 15 nM to 250 nM. The vorinostat concentration range was from 0.25 *μ*M to 5 *μ*M. In all studies, treatment time varied from 4 hours to 72 hours [19] for romidepsin and from 1 hour to 96 hours [21] for vorinostat. Based on the maximum possible time of treatment and the selected concentration in our study, we used a 72-hour treatment. Treatment was administered to the FXS patients cell line GM04025. For all concentration ranges, we analyzed cell viability and determined the presence of* FMR1* mRNA in cells after treatment. Romidepsin did not activate* FMR1* gene expression; moreover, the treatment with high concentrations of the drug led to death of all cells. Thus, preliminary experiments indicated that romidepsin cannot be used as a* FMR1* gene activator in FXS cell lines because there is no detectable level of* FMR1* mRNA after treatment and very high cytotoxicity. In contrast, vorinostat activated* FMR1* gene expression at concentrations 5 *μ*M, but high cytotoxicity was observed.

### 3.3. Activation of FMR1 Expression by Vorinostat Treatment

Cell lines were treated with vorinostat for 72 hours. As a positive control, we treated cells with trichostatin A, a well-known HDAC inhibitor that can activate* FMR1* expression in FXS cell lines as previously reported [10]. Two FXS cell lines and two control cell lines were treated. In GM06895 and GM06865 cell lines, no statistically significant differences in* FMR1* gene expression were noted after treatment with trichostatin A (*P* > 0.05) (Table 1(b)). Regarding vorinostat treatment, only the cell line GM06895 exhibited a significant minor increase in gene expression (*P* < 0.05). In the FXS cell line CPG7, no detectable* FMR1* transcripts were noted in response to vorinostat and trichostatin A treatment (Table 1(b)). The FXS cell line GM04025 reactivated* FMR1* gene expression after treatment with both drugs. However, the differences between the effects of these drugs are not statistically significant (*P* > 0.05).* FMR1* gene transcription levels in GM04025 after treatment with trichostatin A and vorinostat are just 6% and 14% of expression in the control cell line GM06895.

**Figure 1:**
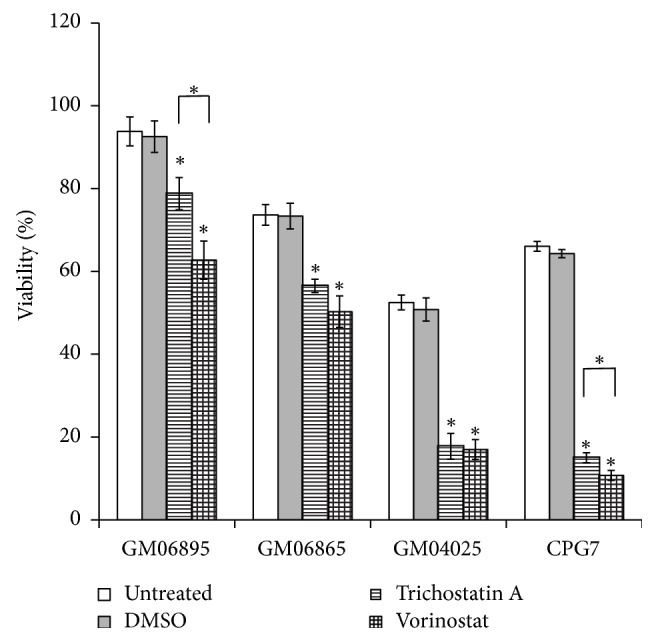
Viability of control and FXS cell lines after treatment with HDACs inhibitors (^*∗*^*P* < 0.05).

All cell lines showed statistically significant decreases in viability after trichostatin A and vorinostat treatment (Figure 1). Vorinostat is significantly more toxic than trichostatin A in all cell lines except GM04025, where the difference is not statistically significant. The viability of control cell lines is increased compared with FXS cell lines. After treatment with HDAC inhibitors, the decrease is not as dramatic as that observed in patient cell lines (Figure 1).

**Figure 2:**
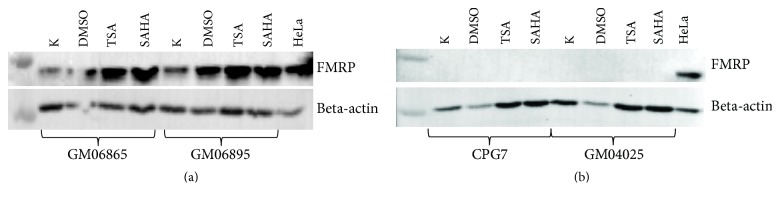
FMRP level of control (a) and FXS (b) cell lines after treatment with HDACs inhibitors.

Western blot analysis revealed a minor increase in FMRP levels in control cell lines after treatment with HDAC inhibitors (Figure 2). Patient cell lines have no FMRP even after treatment with HDAC inhibitors despite the presence of* FMR1* mRNA.

## 4. Discussion

In this research, we studied ability of the FDA approved HDACs inhibitors romidepsin and vorinostat to activate* FMR1* gene expression in FXS cell lines. Treatment with increasing concentrations of romidepsin leads to cell death without* FMR1* gene activation. In contrast, 5 *μ*M vorinostat activated* FMR1* gene expression in the GM04025 FXS cell line. Romidepsin and vorinostat are both inhibitors of class I HDACs. Vorinostat additionally inhibits class II HDACs [11, 12]. Activation of the* FMR1* gene by vorinostat and the absence of activation after romidepsin treatment indirectly indicate that class II HDACs but not class I HDACs play a role in* FMR1* promoter heterochromatinization in the FXS patients cell line GM04025.

Vorinostat reactivates* FMR1* gene expression in the FXS cell line GM04025 at low levels. Similar low levels of reactivation were previously demonstrated with treatment with another HDAC inhibitor trichostatin A [10]. In GM04025,* FMR1* gene activation with trichostatin A or vorinostat produce similar levels of mRNA with no statistically significant differences (*P* > 0.05).

Activation of* FMR1* expression in normal cell lines in response for 5-azadC treatment possibly means that cells in culture exhibit different methylation statuses of the* FMR1* promoter. Two control cell lines had different levels of* FMR1* expression before treatment and consistently the same level after 5-azadC treatment. It is possible that treatment results in a demethylated* FMR1* promoter and maximal gene expression in all cell lines.

The activation of* FMR1* gene expression with DNMT inhibitors is increased compared to activation with HDACs inhibitors. This finding was demonstrated in our research and in previous works [8, 10]. These results suggest that DNA methylation has a priority for gene inactivation. Histone modifications are result of the DNA changes.

The very high cytotoxicity for FXS cell lines and low activating ability make HDAC inhibitors an unviable drug option for FXS therapy development.

Also, it should be noted that other ways of* FMR1* gene silencing were proposed. Transcription of 5′ UTR of* FMR1* gene leads to R-loop formation in GC-rich DNA and its interaction with RNA followed by RNA:DNA hybrid formation. Such structures are proposed to be a trigger for hypermethylation of* FMR1* gene promoter [22, 23]. Thus, further investigation of this effect can lead to the development of a fundamentally new FXS therapy.

## 5. Conclusion

Inhibitors of histone deacetylases are very weak reactivators of the FMR1 gene in Fragile X syndrome cell lines. Some cell lines activate the gene expression at modest level; other cell lines show absence of reactivation. All inhibitors of histone deacetylases are very cytotoxic. These facts make it be not applicable for anti-Fragile X syndrome drugs development.

## Figures and Tables

**Table 1:** Expression of *FMR1* gene in different cell lines depending on treatment (values relative to *FMR1* expression in GM06895, see Materials and Methods).

Cell lines	(a) 5-azadC		(b) HDAC inhibitors			
	Untreated	Treatment	Untreated	DMSO	Trichostatin A	Vorinostat
Control						
GM06895	1.02 ± 0.30	12.09 ± 3.27	1.00 ± 0.11	1.11 ± 0.05	1.36 ± 0.15	1.41 ± 0.08
GM06865	5.57 ± 1.19	10.48 ± 0.22	9.50 ± 2.85	7.36 ± 1.10	9.85 ± 1.86	6.05 ± 2.59
FXS						
GM04025	0	4.97 ± 0.20	0	0	0.06 ± 0.01	0.14 ± 0.07
CPG7	0	0.30 ± 0.05	0	0	0	0
